# Idiopathic primary spontaneous enterolith with intestinal obstruction: A case report with a review of the literature

**DOI:** 10.1016/j.ijscr.2025.111315

**Published:** 2025-04-17

**Authors:** Vidit Dholakia, Suvendu Sekhar Jena, Amitabh Yadav, Samiran Nundy

**Affiliations:** Institute of Surgical Gastroenterology, GI and HPB Onco-Surgery and Liver Transplantation, India

**Keywords:** Enterolithiasis, Intestinal obstruction, Gastrointestinal tract, Case report

## Abstract

**Introduction and importance:**

Enterolithiasis, the presence of stones within the gastrointestinal tract, is a rare condition with an incidence of 0.3 to 10 %. The incidence has increased due to advances in imaging techniques and longer life span of patients. Enteroliths are formed within areas of stasis due to various conditions. These can be primary or secondary, true or false, and can cause obstruction, perforation, or may be asymptomatic. We present an elderly female who had a history of obstruction and who, at operation, had a large stone in her intestine which was causing obstruction but had no distal stricture.

**Case presentation:**

A 58-year-old woman who had diabetes and hypertension was admitted complaining of severe abdominal discomfort, vomiting, bloating, and constipation for 15 days. Diagnostic imaging showed gallstones and dilated small intestines. A large enterolith was discovered in the distal jejunum during surgery and was successfully removed. The patient had a smooth recovery and was discharged on the seventh day post-surgery.

**Clinical discussion:**

Enterolithiasis, first reported by Chomelin, involves stone formation within the intestine due to stasis or altered motility. Clinical presentation varies from asymptomatic to obstruction. Diagnosis relies on imaging, though definitive identification requires stone analysis. Management includes expectant, endoscopic, or surgical approaches, depending on size and associated pathology, ensuring prevention of recurrence.

**Conclusion:**

Enterolithiasis, although rare, has been more frequently diagnosed recently. Large stones can cause obstruction. Identifying their cause can prevent recurrences. Surgery is the primary treatment, but endoscopic techniques could offer less invasive options.

## Highlights

• Enterolithiasis, characterized by intestinal stones, is a rare entity with an increasing incidence due to improved diagnostics and longer life spans.
• Idiopathic large true primary enterolith causing obstruction was identified in a middle-aged woman without predisposing factors or intestinal abnormalities.
• Preoperative imaging indicated stricture, but enterolith was diagnosed intraoperatively. Stone analysis confirmed it was a de novo formation unrelated to gallstones.
• Surgical extraction via enterotomy was performed successfully, along with laparoscopic cholecystectomy. Postoperative recovery was uneventful.
• Enhanced endoscopic techniques like double-balloon enteroscopy hold promise for minimally invasive management of enterolithiasis.

## 1 Introduction

Enterolithiasis is a rare condition and is defined as the presence of stones or concretions within the gastrointestinal tract. It has an incidence of 0.3 to 10 % [1]. The incidence has increased recently due to advances in imaging techniques and the longer life span of patients. They can either be primary (originating within the intestine) or secondary (formed externally and then moving into the gastrointestinal tract). They can be also divided into true (due to precipitation of chyme) or false (formed around insoluble foreign substances like bezoars) [2]. True primary enterolithiasis is often associated with localized stasis, impaired peristalsis, or structural abnormalities within the bowel, leading to the precipitation of bile salts and other substances. In cases where no underlying etiology is identified despite comprehensive evaluation, the diagnosis of idiopathic true primary enterolithiasis is considered. Enteroliths, regardless of type, can cause obstruction, perforation or may be asymptomatically cleared from the intestine [3]. In this report, we describe an instance of intestinal obstruction in a middle-aged woman, which was caused by a primary large enterolith without any obvious identifiable cause. This case report has been reported in line with the SCARE criteria [20].

## 2 Case report

**Fig. 1:**
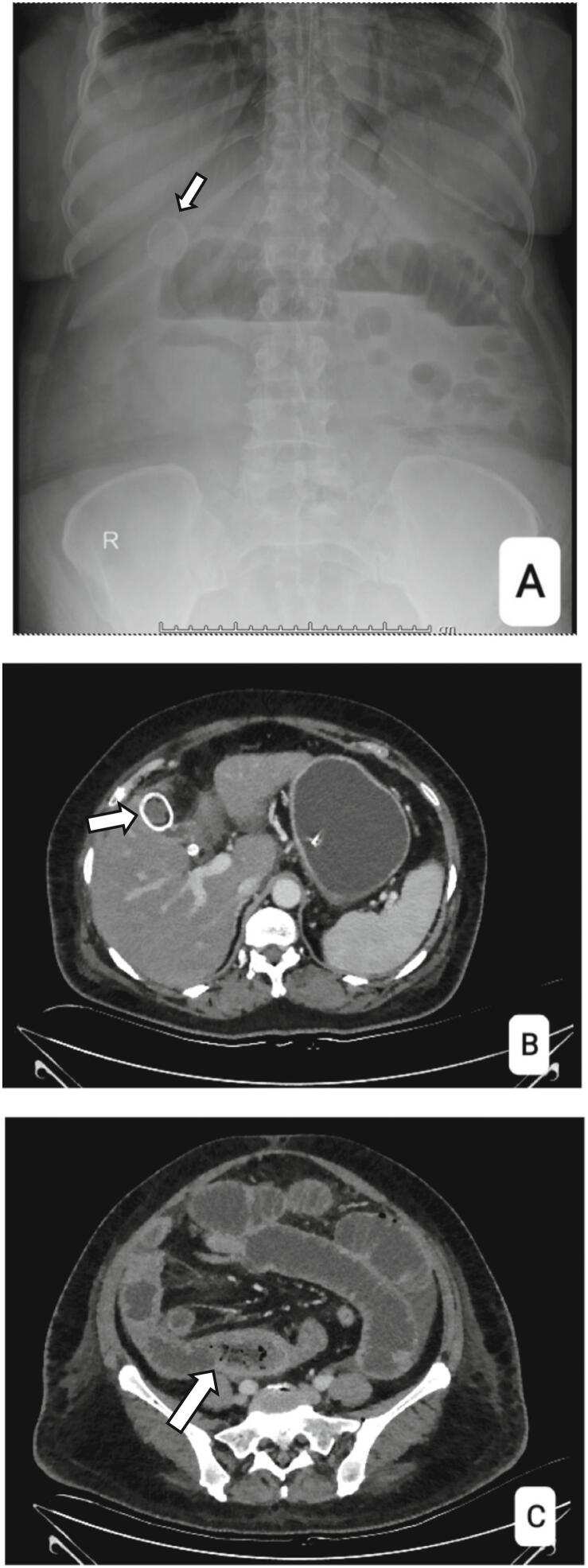
A. X-ray abdomen Erect: Gall bladder calculus and multiple air fluid levels. B. CT Abdomen: Gall bladder calculus. C. Concentric wall thickening with stricture formation in the proximal ileal loops with marked jejunal dilatation. The relevant findings are highlighted by the bold white arrow.

**Fig. 2:**
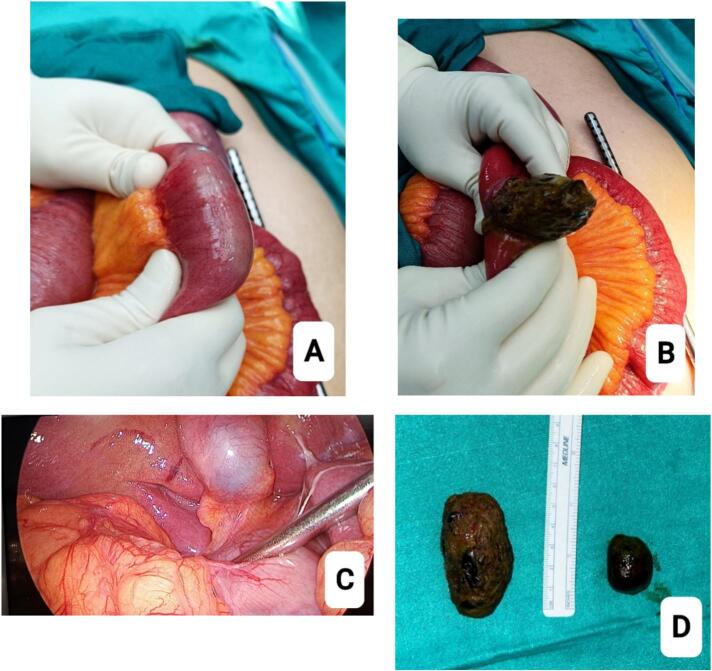
A. Enterolith palpated in small bowel. B. Enterolith delivered via an enterotomy. C. Laparoscopic view of the gallbladder (no bilio-enteric fistula). D. Gallbladder stone specimen with calculus.

A 58-year-old female housewife, who had diabetes mellitus and hypertension, presented to us with complaints of diffuse abdominal pain, multiple episodes of bilious vomiting, abdominal distension and non-passage of stools and flatus for 15 days. On evaluation at a local hospital ultrasound of the whole abdomen showed multiple gall bladder calculi with moderate ascites. Contrast enhanced computed tomography of the abdomen demonstrated dilated small bowel loops with long segment inflammatory thickening of a distal jejunal loop with moderate ascites. The patient was referred to our hospital an erect abdominal radiograph showed a well-defined, calcified, oval shadow in the right upper quadrant suggestive of gall stones with dilated small bowel loops and air-fluid levels. (Fig. 1A) She underwent contrast enhanced computed tomography which revealed concentric wall thickening with stricture formation in the proximal ileal loops with marked jejunal dilatation along with cholelithiasis. (Fig. 1B, C). A diagnosis of intestinal obstruction due to a jejunal stricture was made and surgery was planned. She underwent exploratory laparotomy through a small midline incision and to our surprise we found a 5.5 × 2.5 cm sized enterolith in the distal jejunum which had oedematous bowel walls with dilatation proximal to the enterolith and collapsed loops distally. The enterolith was milked to the proximal non-oedematous bowel and extracted through an enterotomy. The enterotomy was closed transversely after decompressing the proximal bowel loops. No gross bowel wall stricture was found on palpation and on distending the distal bowel with saline. There was no fistulous communication between the gallbladder and bowel and there were no adhesions in the subhepatic region. (Fig. 2) Following the retrieval of the enterolith, the decision was made to perform a laparoscopic cholecystectomy. This approach was preferred to minimize the extent of the incision and reduce the risk of postoperative complications. After primary closure of the midline laparotomy, a 10 mm laparoscopic port was inserted through the closure site, facilitating a minimally invasive cholecystectomy. Patient had a normal postoperative course with a CT scan with oral contrast showing no evidence of leakage, obstruction or strictures. She was discharged on day 7 post operatively.

The enterolith was sent for analysis. It was found to be composed of bile salts (30 %), bilirubin (30 %), biliverdin (20 %) and haematin (20 %), while the gall stones revealed 100 % cholesterol with calcium deposits. Histopathological analysis of the gall bladder showed changes of chronic cholecystitis with focal cholesterolosis.

## 3 Discussion

The French physician Chomelin first reported enterolithiasis after finding a stone in a duodenal diverticulum during an autopsy, in the medical series of Historie de l'Academie Royal [4].Its incidence varies widely from 0.3 % to 10 % and is mostly under reported due to spontaneous passage of smaller stones. The improvement in survival, improved surgical techniques, dietary consumption of calcium and rampant use of acid suppressive therapy may be responsible for the recent increase in incidence [5]. Primary enteroliths, which originate within the intestine, are categorized as being either true or false. True primary enteroliths are created from chyme under normal circumstances and can be further classified into cholic acid and calcium stones. False primary enteroliths, on the other hand, form from insoluble substances in the bowel. These can result from bezoars, the precipitation of substances due to the absorption of their solvents, or the concentration of salts suspended in water [5]. Secondary stones are formed outside and migrate into the gastrointestinal tract with gall stone ileus being the most common causes. Primary enteroliths are formed within areas of stasis like stricture, diverticula, anastomoses, blind loops or intestinal kinks due to adhesions [6]. In our patient, there was no history of prior surgery or tuberculosis. Anatomy, luminal pH, microenvironment along with intestinal pathology, altered intestinal motility and peristalsis play important role in enteroliths formation [7,8]. True primary enteroliths, which are created from substances in the intestine, vary based on their location. Cholic acid enteroliths, which need a lower pH, are typically found in the proximal small bowel. Conversely, calcium stones are located in the distal small bowel [7,8]. The concentration of cholic acid is highest in the duodenum and proximal jejunum (52 % – 84 %) and reduces distally [5]. Similarly, free fatty acids and neutral fat concentration is highest in the proximal bowel (9.6 %) and reduces to 0.6 % distally. Proximal stones are usually calcium free as precipitation of calcium requires an alkaline pH. The stone analysis in our case showed bile salts, bilirubin, biliverdin and haematin as its content while the analysis of the gall stone revealed 100 % cholesterol stone with calcium deposits. The discrepancy in stone compositions confirmed that it was a de novo enterolithiasis. The absence of structural abnormalities and the presence of diabetes mellitus, which is associated with gastrointestinal dysmotility, suggest that impaired intestinal peristalsis may have contributed to enterolith formation in our case. Intestinal stasis, characterized by prolonged luminal transit time, allows for the gradual deposition and aggregation of bile salts and other substances, resulting in enterolithogenesis.

The size and quantity of enteroliths can differ, ranging from a few millimeters up to 10 cm [1]. Clinical presentation varies according to their size, number of stones, associated disease conditions, age of the patient and socioeconomic status. Usually, they are asymptomatic. The symptoms are those of intestinal obstruction, mainly intermittent obstruction also known as ‘tumbling’ obstruction as the enterolith tumbles across the intestine [8]. The presentation may be acute obstruction in case of complete obstruction and rarely bowel perforation. Our patient presented with symptoms of intestinal obstruction without any similar history in the past.

A proper history and physical examination are of paramount importance. Blood pictures may reveal leukocytosis and anemia due to pressure of the enterolith on the intestinal mucosa [2]. As an initial investigation, abdominal roentgenograms are helpful in diagnosing enteroliths in a third of cases [9]. However the absence of calcium in proximal cholic acid stones leading to radiolucency is a limitation. Few of the important features are their dense rim with a pale core, “coin-end-on” appearance and mobility on serial examination [10]. Computed tomography with oral contrast provides information like the number, size and location of the stone along with associated intestinal pathology if any. Special attention should be paid to the gall bladder to rule out gall stone ileus [2]. However, a definitive diagnosis of the site of origin can only be made by removing the stones and its subsequent analysis [5]. Computed tomography in our case showed suspicion of intestinal stricture and did not diagnose the stone preoperatively. On exploration, there were no adhesions, small bowel diverticulum or a Meckel's diverticulum. Despite our best efforts, we could not find the cause of such a large enterolith and the probable explanation can be slow intestinal motility due to diabetes mellitus and old age.

The formation of enteroliths can serve as an initial indication of a disrupted intestinal structure, and rectifying this is necessary to avoid the creation of future stones. The management depends upon the presentation of the patient. For stones of size less than 2 cm in whom spontaneous passage is possible, expectant management in the form of hydration, electrolyte correction, nasogastric aspiration should be adopted while for large stones greater than 2 cm spontaneous passage is unlikely and the stones should be removed [11].In case of strictures, endoscopic dilatation of stricture along with retrieval of stones can be tried [12,13].The use of lithotripsy has also been described [14,15].However, majority of obstructing enteroliths require surgical management. Various options are available like fragmenting the stone and milking it into the colon or milking the stone proximally into non-edematous bowel and retrieving it via an enterotomy [16,17]. Although most of the cases described in literature are managed via the open approach, laparoscopy can also be tried as shown by Jones et al., Shah et al. [18,19] The patient mentioned in this case report was managed in a tertiary care private practice setting.

### 3.1 Strengths

This is a unique case report of spontaneous enterothiasis presenting with obstruction, suspected to be Gall stone ileus and operated with Hybrid (Small incision laparotomy f/b Laparoscopic) approach.

### 3.2 Limitation

Follow up after discharge was not available for this patient.

## 4 Conclusion

Enterolithiasis are rare but their incidence has increased recently. Although usually asymptomatic, large stones can cause intestinal obstruction. An effort should be made to find out the cause of enterolithiasis as its correction can prevent future recurrences. Currently, surgery is the mainstay of management but the advances in endoscopy like double balloon enteroscopy can open up a new arena in managing such cases less invasively.

## Consent to publish statement

Written informed consent was obtained from a legally authorized representative(s) for anonymized patient information to be published in this article.

## Study approval statement

Ethical approval is not required for this study in accordance with local or national guidelines.

## Funding

This study was not supported by any sponsor or funder.

## Author contribution

Vidit Dholakia: Writing - Original Draft.

Suvendu Jena: Writing - Review & Editing.

Amitabh Yadav, Samiran Nundy: Writing - Review & Editing, Supervision.

All the authors reviewed the manuscript.

## Guarantor

Vidit Dholakia.

## Research registration number

Not required at our institution, i.e., Sir Ganga Ram Hospital.

## Declaration of Generative AI and AI-assisted technologies in the writing process

During the preparation of this work the author(s) used ChatGPT in order to improve readability and language. After using this tool/service, the author(s) reviewed and edited the content as needed and take(s) full responsibility for the content of the publication.

## Conflict of interest statement

The authors have no conflicts of interest to declare.

## Data Availability

All data generated or analyzed during this study are included in this article [and its supplementary material files]. Further enquiries can be directed to the corresponding author.
